# Searching for cancer vulnerabilities amid genetic chaos

**DOI:** 10.1186/s13059-017-1283-2

**Published:** 2017-08-03

**Authors:** Michael R. Speicher

**Affiliations:** 1 Institute of Human Genetics, Medical University of Graz, Harrachgasse 21/8, A-8010 Graz, Austria; 2 BioTechMed-Graz, Graz, Austria

**Keywords:** Cancer genomes, Intratumor heterogeneity, Tumor microenvironment, Driver genes, Non-coding genomic regions, Liquid biopsies, Cancer immunology, Immunotherapies, Bioinformatics

## Abstract

A meeting report on the Third European Association for Cancer Research Conference on Cancer Genomics, held at Churchill College, Cambridge, UK, 25–28 June 2017.

## Introduction

Cancer is an extremely complex disease. Recent large-scale studies have revealed fascinating insights into the genetic make-up of multiple tumor entities, but numerous challenges remain. Many of these challenges were addressed at the biannual European Association for Cancer Research Conference on Cancer Genomics, held at an inspiring venue in Cambridge (UK) next to the park where Francis Crick and James Watson relaxed and discussed their theories.

The format of this meeting is deliberately kept small, with a maximum of 220 participants to provide an intimate setting for intensive scientific interactions. Most speakers presented unpublished data, which provoked lively discussions about the latest developments during lunch breaks, dinners, and at the crowded poster sessions. The limited space of this meeting report makes it impossible to summarize every presentation, so I apologize for any omissions. However, here I provide my personal highlights and views of important themes.

## The hunt for new cancer driver genes and mutations

Driver mutations are particularly important for cancer initiation and progression, as they provide cells with a selective growth advantage. Few driver events are currently known, and Gad Getz (Broad Institute, USA) presented on the subject of ambitious efforts to generate a complete catalog. He noted that most cancer driver genes occurring in more than 20% of tumors are probably known, whereas the discovery of new cancer genes mutated in fewer than 10% of tumors is steadily growing.

Núria Lopez-Bigas (Institute for Research in Biomedicine, Barcelona, Spain) supported these notions. She estimated there to be 3.1 driver point mutations per tumor, and 4.3 driver events per tumor. According to her, we can identify at least one driver per tumor in 91% of cases, but we do not know how many drivers we are missing.

There are also unknowns for well-established driver genes. For example, Eran Kotler (Weizmann Institute of Science, Israel) highlighted that whereas six hotspot mutations comprise 30% of all mutations in *TP53*, the most frequently mutated gene in human cancer, the remaining 70% is made up of about 10,000 different mutations, many of which have unknown consequences.

## New hunting grounds for driver genes: non-coding regions

Recognition that there are “missing” driver genes has increasingly extended search efforts to non-coding regions. Gad Getz reported a screen of breast cancer genomes that revealed several significantly mutated promoters and long non-coding RNA genes. Only a few cohorts are large enough to discover drivers occurring with low frequency; therefore, metacohorts are being generated to increase the statistical power. To this end, both Gad Getz and Núria Lopez-Bigas referred to the Pan Cancer Analysis of Whole Genomes network, in which the whole genome sequences of more than 2800 tumor/normal pairs from 37 different tumor types were generated. Núria Lopez-Bigas highlighted that chromatin organization, DNA accessibility, and replication timing significantly influence the rate of somatic mutations across the genome. She estimated that 18% of driver mutations are in non-coding regions and occur in 33% of tumor samples.

## Intratumor heterogeneity and the community of clones

Intratumor heterogeneity (ITH) adds an additional level of complexity to these multifaceted patterns of mutations. Charles Swanton (The Francis Crick Institute, UK) has conducted spatial and temporal genomic analyses of samples from 842 lung cancer patients within the TRAcking Cancer Evolution through therapy (TRACERx) study. He reported that mutational processes are not uniform among the branches of cancer clonal trees and, as the disease evolves, trunks shrink and branches grow. This is reflected in the distribution of clonal mutations, which make up >30% of mutation burden at diagnosis but <10% of mutation burden at death.

Within the context of increasing insights into the molecular features of ITH, Carlos Caldas (Cancer Research UK (CRUK), Cambridge Institute, UK) repeatedly stressed that tumors should be viewed as symbiotic communities of clones that are maintained during metastasis.

The significance of ITH was further developed by Stefan Dentro (Sanger Institute, UK), who presented data from an International Cancer Genome Consortium pan-cancer analysis of 2778 whole genomes from 39 histologically distinct types. This analysis found pervasive subclonality in most tumors, with driver mutations frequently occurring in subclones.

## Chromosomal instability and somatic copy number alterations

Several speakers emphasized the importance of chromosomal instability and somatic copy number alterations. For example, Charles Swanton recalled the pioneering work of Richard Goldschmidt (1878–1958), who believed that large changes in evolution were caused by large “macromutations”. According to Swanton, macromutations might include genome doubling, a common and often early event in lung cancer that appears to accelerate cancer genome evolution.

James Brenton (CRUK, Cambridge Institute, UK) meticulously dissected the copy number landscape of high-grade serous ovarian cancer using parameters such as breakpoint number and their locations. He described the exciting finding that multiple copy number signatures exist within these cancers. Importantly, these signatures predict therapy response and thus represent new tools for precision medicine in this tumor entity.

## Liquid biopsies

Options to monitor tumor genome evolution by non-invasive liquid biopsies, i.e., circulating tumor cells (CTCs) or circulating tumor DNA (ctDNA) were intensively discussed.

Caroline Dive (CRUK, Manchester Institute, UK) presented a 16-segment “copy number alteration signature” of response to therapy derived from CTCs, which allows clinicians to determine whether or not a patient is chemosensitive before treatment. However, identifying epithelial cell adhesion molecule-negative CTCs (i.e., highly aggressive and invasive CTCs that do not express epithelial proteins) remains problematic, and efforts to improve CTC detection based on cellular markers are underway.

Nicholas Turner (Institute of Cancer Research, UK) reported that ctDNA is detectable in >90% of patients with metastasized breast cancer. However, the ctDNA detection rate varies greatly in certain breast cancer subtypes, such as triple-negative, or ER^+^ and HER2^–^. He observed that detecting ctDNA can predict relapse several months earlier than using clinical parameters.

In presentations from our own group, Jelena Belic (Medical University of Graz, Austria) showed that, in men with prostate cancer, it is possible to identify the transition from adenocarcinoma to neuroendocrine prostate cancer using ctDNA because of the emergence of a new clone. In my own presentation I described how expressed genes in the cells, which release their DNA into the circulation, can be inferred by whole-genome sequencing of plasma DNA.

## Modeling cancer

Disease models that reliably reflect the characteristics of their tumors, and which can be used as therapeutic predictive models, are instrumental in the development of novel treatment options. Several strategies were presented.

Caroline Dive showed that CTC-derived patient explants reliably mirror donor patient response to chemotherapy, and can be used to explore drug combinations depending on the genomic characteristics of the tumor.

Alejandra Bruna (CRUK, Cambridge Institute, UK) demonstrated that patient-derived tumor xenografts capture the most fundamental features of breast cancers (for example heterogeneity), and—importantly—that drug response in vitro predicts drug response in vivo. Chris de Witte (University Medical Center Utrecht, Netherlands) presented an ovarian cancer organoid biobank, which currently comprises 67 organoids from 39 patients.

Olli Kallioniemi (SciLifeLab, Sweden) demonstrated examples of individualized systems medicine based on patient-derived primary cancer cells to conduct integrative ‘omics and functional drug testing, and to identify molecularly defined subgroups of patients.

## Genomic and immune landscapes in cancer and immunotherapies

Improved treatment options require a better understanding of immune landscapes in cancer. Presentations on several exciting studies addressed this topic.

Francesca Ciccarelli (Francis Crick Institute, UK) reported that synchronous colorectal cancers are genetically independent, and follow independent evolutionary pathways. Patients with these tumors carry damaging germline single nucleotide polymorphisms in immune genes. These may cause an inflammatory microenvironment in the gut, and hence predispose patients to developing several independent tumors.

Ido Amit (Weizmann Institute of Science, Israel) presented on immunology in the age of single cell genomics. Since immune cells are plastic and dependent on their environment, interpretation of results therefore requires knowledge of both the location and time of origin of these cells. He presented a single-cell pipeline with a throughput of 10,000–20,000 single cells per day, which contributes to improved definitions of both “cell type” and “cell state” and has enabled rare cell populations to be identified. As an example he showed a recently identified protective microglia type associated with Alzheimer’s disease.

Yardena Samuels (Weizmann Institute of Science, Israel) showed data from a pipeline for filtering cancer exome databases to identify novel antigens. This pipeline identified fewer neo-antigens than expected, and the number did not correlate with the number of mutations. By employing sophisticated methods to find neo-antigen-specific tumor-infiltrating lymphocytes, she showed fascinating images of melanoma cells being killed by these autologous lymphocytes.

Based on somatic profiles of 2433 breast cancer samples, Carlos Caldas emphasized that each genomic subtype has a distinctive immune tumor microenvironment and immune editing pattern. He showed an impressive dataset implying that therapies shape the tumor genome and microenvironment. As the immune tumor microenvironment and T-cell receptor clonal distribution can differ between metastases, the shape of metastatic phylogenies is highly complex and varied.

Ton Schumacher (Netherlands Cancer Institute) lectured on T-cell recognition in human cancer. Employing RNA-seq data, a map of mutations was generated and potential epitopes for each mutation were evaluated. He explained the importance of understanding T-cell exhaustion states in human cancers, because such knowledge may help to kick these cells back into action. However, the subset of T cells that are tumor-reactive are very small. He and Marian Burr (Peter MacCallum Cancer Centre, Australia) presented independently generated data demonstrating that CMTM6 is a novel regulator of PD-L1 in multiple tumor types. Although the PD-1/PD-L1 axis is known to be a key suppressor of anti-tumor immunity, the features determining PD-L1 protein levels on the cell surface were unknown. Both researchers have now shown that PD-L1 expression is impaired in human tumor cells when CMTM6 is downregulated.

Sergio Quezada (University College London, UK) demonstrated his efforts to map the immune and antigenic landscape of cancer to develop novel immunotherapeutic strategies. Tumors have an altered balance of T-cell types and an increased proportion of regulatory T cells that favor tumor growth and immune escape. For example, CD25 is highly expressed on regulatory T cells, and, in mouse models of several transplantable tumor cell lines, an optimized anti-CD25 antibody in combination with anti-PD1 resulted in impressive response rates.

## Potential treatment options

Caitlin A. Nichols (Dana-Farber Cancer Institute, USA) used elegant strategies to identify single nucleotide polymorphisms in essential genes in loss of heterozygosity regions within cancer genomes. She then used CRISPR-Cas9 to deactivate allele-specific genes, and showed that if only the CRISPR-sensitive allele is left, the cell may die because of loss of heterozygosity.

René Bernards (Netherlands Cancer Institute) presented a “one–two punch model” for cancer therapy: the first punch induces vulnerability, whereas the second punch targets this induced vulnerability. He presented examples including treatment of RAS-mutant tumors with histone deacetylase inhibitors; these increase levels of reactive oxygen species and are thus toxic for the cells. Another example was sequential dosage with BRAF inhibitors and histone deacetylase inhibitors to improve outcomes in the treatment of melanoma patients.

## Handling large-scale data

Current cancer genomics research is generating large and complex datasets, and many speakers stressed the importance of rigorous statistics and bioinformatics. Numerous tools were presented, which cannot all be listed here. A seminal example was the cloud computing-based FireCloud (https://software.broadinstitute.org/firecloud/) presented by Gad Getz; an open-source and scalable analysis platform for collaborative science.

## Conclusions

Carlos Caldas, one of the organizers, referred to this meeting as the “premier cancer genomics meeting in Europe”, and this is not an overstatement. It was fascinating to hear the latest updates on the complexities of human cancer, while at the same time observing the efforts to master these challenges. Furthermore, promising therapeutic strategies are emerging from the chaos of cancer genomes.

## Abbreviations

CRUK: Cancer research UK
CTC: Circulating tumor cell
CtDNA: Circulating tumor DNA
ITH: Intratumor heterogeneity

